# Supplementary material for: Can women’s reports in client exit interviews be used to measure and track progress of antenatal care services quality? Evidence from a facility assessment census in Malawi
Source: PLoS One. 2023 Jul 31;18(7):e0274650. doi: 10.1371/journal.pone.0274650 (PMC10389737; doi:10.1371/journal.pone.0274650)
Supplement: Attachment — Submitted filename: Editor letter & Reviewers comments .docx [file pone.0274650.s001.docx]

Dear Editor

# Cover Letter for Manuscript submission entitled: “Can women’s reports in client exit interviews be used to measure and track progress in antenatal care quality? Evidence from a facility assessment census in Malawi”

On behalf of my co-authors and I, I write to submit the aforementioned manuscript which was previously submitted as “Within-consultation comparisons of antenatal care quality assessments via patient exit interviews and expert direct observations in Malawi”.

The manuscript was initially given an open rejection. Having gown through the comments and suggestions raised by the reviewers, we took a step back to rethink and redo the analysis. We further also had to rewrite and restructure the manuscript in a way that complied with Plos one guidelines but also make the subject more clear and easier to understand. All thanks to the reviewers for their thorough work in reviewing and bringing out the issues in the previous manuscript.

We therefore, hope that we have done a good job in addressing the issues which were previously raised. The responses to the reviewers is attached to this letter below.

Thank you for taking time to consider this manuscript in your journal.

Sincerely,

Martina Mchenga

**Response to reviewers**

1. **Reviewer 1**

Reviewer #1: This is an important study that explored the gap between expert's direct observations and patient exit interviews. However, significant improvements are needed in the following points, thus "open reject" is recommended.

**Response**- Thank you so much, we took time to address the comments raised and we feel that the new manuscript is a better and improved version of the previous submission and we would like to thank you for your inputs in it.

1. Plosone's review screening criteria state that the manuscript should follow the reporting guidelines. However, this paper does not follow them (no description of research objectives or research design for examples). So, the authors need to rewrite it according to one of the reporting guidelines.

**Response**- Thank you, this has been done. Check the methods section on pages 4-7

2. The authors stated that the high maternal and neonatal mortality rates in Malawi were due to the weak effect of antenatal care. However, most maternal deaths occur during delivery, and the risk of neonatal deaths is highest in the first 24 hours after delivery. Thus, ANC is not the leading cause of death.

**Response**-This is true. However, the argument is that instead of just looking at the number of visits, it is also important to look at the content/ quality of care that the women are receiving. This has been raised by WHO and other researchers.

3. Lines 89-95 on p. 5 should be written in the discussion.

**Response-**Thank you. This has been moved.

4. It is hard to understand what "quality of care" is targeted in this study. From the title and introduction, I can guess that "appropriateness of counseling" was the targeted "quality of care" in this study. However, the variables used to examine "quality of care" include the content of the care received (iron pills, etc.). The definition of targeted quality of care of this study should be clearly stated, and the title, introduction, and methods should be appropriate for them.

**Response**-This is a great suggestion. Thank you. The quality of care targeted for this study Is the interventions that women are expected to receive during an ANC consultation. These are based on WHO guidelines and Ministry of health guidelines. However, we could not include everything because of data limitation. For the purposes of this study we included 6 indicators of quality which were captured in both the direct observation guide and the exit interview questionnaires. These include; whether the provide prescribed or gave iron/folic acid tablets, whether the provider prescribed or gave malaria tablets, whether the provider informed the woman about iron tablets side effects, whether the provider informed the woman about pregnancy related complications, whether the provider informed the woman what to prepare for delivery and whether the provider discussed the importance of nutrition in pregnancy.

All this has been explained in the methods section pages 3-4.

5. Table 2. Is "mean" in the table an error for "percentage"? Also, what does the standard deviation of “education background” indicate?

**Response**-Table 2 has been revised and its now Table 1. We have used percent and confidence intervals for easy interpretation and understanding.

6. Several studies have compared the gap between direct observation and exit survey results. So, the present study results need to be compared with those studies and described in the discussion section. For example, the following paper is of a study with a similar method to the authors'.

Onishi J, Gupta S, Peters DH. Comparative analysis of exit interviews and direct clinical observations in pediatric ambulatory care services in Afghanistan. Int J Qual Health Care. 2011 Feb;23(1):76-82. doi: 10.1093/intqhc/mzq074. Epub 2010 Dec 3. PMID: 21131382.

**Response-**Thank you so much for suggesting the above article. We have included other study findings and also the study suggested. Check the discussion section

2. **Reviewer 2**

**Overall comment**

Overall, the authors discussed the important topic of the measurement of the quality of antenatal care in Malawi. They used the data of the 2013-14 Malawi Service Provision Assessment to conduct the descriptive analysis of the ANC quality indicators captured by direct observation and women’s self-report in existing interviews. The authors need to elaborate more on the materials and methods section to provide enough detail to allow other investigators to fully replicate the study. The level of education was factored in the analysis, but the other potentially associated factors, such as the parity, may be worth being explored. There was a general lack of interpretation of results regarding the discrepancies in the rating of the quality of ANC reported by the two different methods. The authors argue the strengths and weaknesses of direct observation and women’s self-report in existing interviews in discussion but not entirely based on the results. It would be great if the authors could add evidence and implication for policymakers, including how to mitigate the potential biases in reporting, based on the findings of their study.

**Response**-Thank you so much for bringing this up. We indeed agree with every suggestion which has been made in this comment. And because of this, we have tried to redo the whole analysis and rewrite the paper in a clear and logical way. See below the specific areas which we have changed based on the comments.

Please find my comments below.

**Abstract**

- Line 27: The 2013-14 Facility Census should be the 2013-14 Malawi Service Provision Assessment.

**Response-**This has been revised

- Line 27: Please explain the methods used to analyze the data.

**Response**-We have added this to the abstract

- Line 36: It is recommended to add the interpretations of the results. i.e., how the discrepancies between the two methods occurred and potential biases.

**Response**-Thank you so much for this. We have added the recommendation in the conclusion section of the abstract.

**Introduction**

- Overall, the introduction seems lengthy. Please provide only background necessary to put the manuscript into context and avoid multiple sections.

**Response**-Thank you for this commend. We indeed agree with this observation, we have therefore, rewritten and shorten the introduction

- Line 48: Considering potentially diverse situations in health care systems in LMICs, it is better to specify the context. In the literature review, the author mainly discussed African countries except for one study in China (line 170).

**Response**-We have taken time to rewrite the background section and include studies from other countries besides African countries. Check page 3, paragraph 2.

- Line 86: The traditional prioritization of direct observations as the gold standard. Please add references and explain assessment systems and methods for ANC services used specifically in Malawi.

**Response**-Thank you. This has been revised, check page 2, the last paragraph

- Lines 89-95: “Our study adds to the existing literature in at least two ways: first, our data is nationally representative…” should be cut or moved after explaining the data used in this study, or to the discussions.

**Response**-Yes, we have done that

- Lines 96-98: these sentences explaining the structure of the manuscript can be deleted, and the author should follow the journal format.

**Response**-Thank you. We have done as suggested

- Lines 104-105: “Despite these costs and potential biases, most researchers consider direct observations to be the yardstick for measuring the quality of care. Please add a reference.

**Response**-Reference have been added where they were missing.

- Lines 132-134: You explain the concerns that patients may lack the health literacy to capture and reflect accurately the clinical content of the consultation, especially among women with low education, referring to DHS data. I am not sure if DHS can provide evidence on the association between low education and the lack of health literacy to capture and reflect accurately the clinical content of the consultation in Malawi. If other sources are available, please refer.

**Response**-Check on page 12. We decided to test for factors that are associated with agreement between what women reported in the exit interviews and what was observed in the consultation by an expert. We felt this kind of analysis made sense as it would uncover what women perceived was the quality of care they received. For this analysis, education was not significant.

- Lines 156-157: “Relatively few studies consider whether self-reported data are appropriate for measuring maternal healthcare quality, and LMICs are underrepresented in this literature” Add a reference/

**Response-**We have added references, check the introduction section

- Line 159-160: determining quality in developing than in developed country health systems.
- “Developing” and “Developed” countries should be LMICs/HICs.

**Response-**Thank you so much for this. We have used LMICs/HICs throughout the manuscript

- Objectives of the study should be mentioned at the end of the introduction.

**Response**-Thank you, this has been revised.

**Materials and Methods**

- Overall, the Materials and methods section needs to be structured well to provide enough detail to allow other investigators to fully replicate the study. It is difficult to follow the study design, materials, analysis etc. used in this study.
- Please specify study design, settings when data collection for the 2013-14 Malawi Service Provision Assessment was done.
- How health workers were selected, how health facilities were selected for observations, how patients were selected for exit interviews? Any eligibility criteria used for selecting health facilities and patients?
- Who are surveyors for direct observations?
- Lines 220-226: “Measuring quality of care, compared to quantity, has many challenges. Quality is multi- dimensional, with three separate elements of care all contribute to quality: structure, process, and outcome …” Challenges are not explained well, and these should be covered in the introduction, not in the methods.
- Lines 220-232: Quality is multi-dimensional, with three separate elements of care all contribute to quality: structure, process, and outcome, and the authors focused on the process in this study, mentioning process is one of the more important components of health care quality. Please elaborate more on this with justification for why you only focused on process does not structure and outcomes.
- Line 232: Process predicts outcomes. Please explain this based on the evidence on ANC.
- Line 246: The ideas of sensitivity and specificity are difficult to understand. Please add a reference if you used to conceptualize these.
- Lines 267-258: Please check “ROC values yield proportions that start at 0 (no accuracy) and move to 1.0 (when classification is perfect) to 0 (zero accuracy)”. It can be a typo.
- Please explain social and demographic characteristics variables included in the analysis (education, age, parity, type of residence etc.). I guess parity may have an impact on the results of both direct observation and patient exit interviews. Data stratified by education status are included in the results section, so it should be explained in the methods section.
- It is unclear what kinds of analyses were made. Please explain in the methods.
- Explain what kinds of the questionnaire is used for the patient exist interview.

**Response**-We would like to thank the reviewer for this comprehensive comment. We have taken time to rewrite the whole methodology section to address all the concerns raised. Check pages 4-7

**Results**

- Parity may have an impact on the results of both direct observation and patient exit interviews. Are there any analyses considering these potential covariates apart from education?

**Response**-Yes, very true. Check the new analysis on page 12.

- Table 3 shows that sensitivity varied according to years of education. Why do you compare only no education and secondary or higher? The analytical methods need to be explained in the previous section.

**Response**-This has been revised, the methods have been explained in the methods section and other analysis added

- Lines 319-321: “These results are, however, regarded as tentative and exploratory because these differences were large but imprecisely estimated and not statistically significant” Please elaborate more on this sentence. What do you mean by tentative and exploratory? Did you check the significance statically?
- Lines 325-325: This is the interpretation of results, and please move these to discussions.

**Response**-The results section has been rewritten and the points raised addressed.

**Discussions**

- Overall, there was a general lack of interpretation of results regarding the discrepancies in the rating of the quality of ANC reported by the two different methods. The authors argue the strengths and weaknesses of direct observation and women's self-report in existing interviews in discussion but not fully based on the results. It would be great if the authors could add evidence and implication for policymakers, including how to mitigate the potential biases in reporting, based on the findings of their study.
- line 334-335: “In Malawi, over 95% of women have access to antenatal care with skilled personnel, yet maternal and child health outcomes remain disappointing.” Please add a reference.
- Lines 344-347: “This interpretation would also align with the tentative evidence that suggests that specificity is higher amongst the least educated, implying that the tendency towards courtesy bias would be more pronounced amongst lower SES groups as the literature has found”. Spell out SES and add a reference about the association between SES and tendency toward the courtesy bias.
- Lines 358-360: “Looking at the questionnaire, it was clear that the items with high sensitivity had retained medical terms that may not be widely understood, for example, counselling of iron side effects and danger signs for complications in pregnancy." Please explain the questionnaire used in the method.
- Lines 368-370: Low- and middle-income countries should be LMICs.
- Lines 400-403: “we found evidence of deficiencies in both specificity and sensitivity, with tentative evidence that sensitivity had a positive relationship with education while specificity had a negative relationship with education." Again, I recommend you explore other variables related to the sensitivity.

**Response**-Thank you so much for this. We took time to rewrite the discussion section as per your suggestion. Please check the discussion section on page 14
