# Supplementary material for: Can women’s reports in client exit interviews be used to measure and track progress of antenatal care services quality? Evidence from a facility assessment census in Malawi
Source: PLoS One. 2023 Jul 31;18(7):e0274650. doi: 10.1371/journal.pone.0274650 (PMC10389737; doi:10.1371/journal.pone.0274650)
Supplement: Attachment — Submitted filename: Review report on Client exit interviews.docx [file pone.0274650.s002.docx]

**Manuscript ID:** **1075691**

**Topic: Can women’s reports in client exit interviews be used to measure and track the progress of antenatal care quality? Evidence from a facility assessment census in Malawi**

**Handling Editor: Dr. Sohel Saikat**

Thanks very much, Dr. Sohel Saikat, for giving me the opportunity to review and give my comments. My thanks also go to the authors of the manuscript for their interest to deal with this interesting topic addressing the issue of “Can women’s reports in client exit interviews be used to measure and track the progress of antenatal care quality? Evidence from a facility assessment census in Malawi” which are the areas that need more research to cross-check, to address, and to come up with convincing shreds of evidence on reports in client exit interviews be used to measure and track the progress of antenatal care quality. Given all information in mind, the title is impressive, and the way the authors synthesize and present the figure is also appreciated. However, the tittle and the findings are not inline, which means there are mismatch between the topic and the figures stated in the result part. I have seen the whole document thoroughly and based on that I have some comments, and questions to be addressed by the authors before the manuscript will publish.

**Abstract**

The conclusion section of the abstract should be very conclusive and the recommendation also included under it. Please, include the recommendation within the conclusion.

**In the ethical consideration**

You stated that, “….However, we did obtain approval from the National Directorate of Public Health to use the data in our study”. Would you kindly write the approval reference number taken from the stated directorate? Please, put the reference number to increase the credibility of the data to be taken from the stated directorate.

**In the methods**

The methods section was not appropriately organized. Please, revise this part.

**In the result and discussion**

In Table 1, the variables parity and provider-used visual aids were not well calculated, and a single category was reported. Here my concern is, why a single category was reported? Please, report all clatagories.

The discussion is poorly discussed. Please, compare the figures with other findings thoroughly and make them strong as possible.

What are your recommendations? Based on the result and the conclusion drawn from your study, you are obliged to recommend for the concerned stakeholders to tack corrective measures.
