# Supplementary material for: Can women’s reports in client exit interviews be used to measure and track progress of antenatal care services quality? Evidence from a facility assessment census in Malawi
Source: PLoS One. 2023 Jul 31;18(7):e0274650. doi: 10.1371/journal.pone.0274650 (PMC10389737; doi:10.1371/journal.pone.0274650)
Supplement: Attachment — Submitted filename: Rebuttal Letter.docx [file pone.0274650.s004.docx]

Bereket Yakob, Ph.D.
Academic Editor
PLOS ONE

11/07/2023

# Dear Editor

# Thank you for inviting us to submit a revised draft of our manuscript entitled, " Can women’s reports of client exit interviews be used to measure and track progress of antenatal care quality? Evidence from a facility assessment census in Malawi” to PLOS ONE. We also appreciate the time and effort you and each of the reviewers have dedicated to providing insightful feedback on ways to strengthen our paper. Thus, it is with great pleasure that we resubmit our article for further consideration. We have incorporated changes that reflect the detailed suggestions you have graciously provided. We also hope that our edits and the responses we provide below satisfactorily address all the issues and concerns you and the reviewers have noted.

To facilitate your review of our revisions, the following is a point-by-point response to the questions and comments delivered in your letter dated 9 July, 2023.

A. **Abstract**
1. Define what you meant by “objective” and “subjective” measures in the methods section. Were there any cut-off points used?

**Response**- we acknowledge the potential confusion surrounding the terms "objective" and "subjective" measures, so in our study, we opted to use alternative terminology. Specifically, we employed the terms "observable and concrete measures" to refer to aspects that are externally verifiable and tangible, for example, prescription of medication and "measures related to counselling" to denote variables associated with information sharing between the provider and client, for example, provider counselled the client on iron tablets side effects.

There was no cut-off points used, those words were to describe the interventions

2. include statistical figures in the results section of the abstract. The readers need to know what you meant by “high reporting accuracy” and the validity criteria—same comment for reliability.

**Response-** Thank you for this suggestion. We have included the statistics in the abstract.

B. **Main body**
1. **Introduction**

One of the study's aims was to uncover factors associated with agreement between women’s self-reports and expert direct observation reports. However, this was not presented in the abstract. Please present statistical summaries in the results section of the abstract.

**Response-** we have rephrased that aim, see below. What we did was to stratify the agreement measure by the social demographic characteristics of the women. Significance was determined by the chi-squared tests. These results were not the main goal and that’s why we did not include the statistics in the abstract but rather list factors that increased the likelihood of reporting reliability based on the chi-squared test and the associated p-value.

*Using heterogeneity chi-squared tests, the study also examines whether reporting reliability varies significantly by individual and facility level characteristics.*

2. **Methods**

Page 6 Variables of Interest: Define the subjective and objective measures (factors) as you used the terms in the abstract. The other option is for the authors to remove the terms from the abstract.

**Response**- We have removed the terms subjective and objective measures from the abstract and throughout the paper to avoid the confusion.

Thank you once again!

Sincerely,

Martina Mchenga, PhD
